# Are head-down tilt bedrest studies capturing the true nature of spaceflight-induced cognitive changes? A review

**DOI:** 10.3389/fphys.2022.1008508

**Published:** 2022-12-13

**Authors:** Irén Barkaszi, Bea Ehmann, Borbála Tölgyesi, László Balázs, Anna Altbäcker

**Affiliations:** Institute of Cognitive Neuroscience and Psychology, Research Centre for Natural Sciences, Budapest, Hungary

**Keywords:** cognitive functions, spaceflight, microgravity, head-down tilt bedrest, behavioral performance, astronauts, bedrest, brain

## Abstract

Although a number of studies have examined cognitive functions in space, the reasons behind the observed changes described by space research and anecdotal reports have not yet been elucidated. A potential source of cognitive changes is the cephalad fluid shift in the body caused by the lack of hydrostatic pressure under microgravity. These alterations can be modeled under terrestrial conditions using ground-based studies, such as head-down tilt bedrest (HDBR). In this review, we compare the results of the space and HDBR cognitive research. Results for baseline and in-flight/in-HDBR comparisons, and for baseline and post-flight/post-HDBR comparisons are detailed regarding sensorimotor skills, time estimation, attention, psychomotor speed, memory, executive functions, reasoning, mathematical processing, and cognitive processing of emotional stimuli. Beyond behavioral performance, results regarding brain electrical activity during simulated and real microgravity environments are also discussed. Finally, we highlight the research gaps and suggest future directions.

## Introduction

To ensure the success of human space exploration, the risk factors endangering the performance of astronauts must be fully evaluated. Indeed, during spaceflight, the human body is exposed to various space-related stress factors, such as microgravity, cosmic radiation, elevated CO2 levels, altered light exposure, increased ambient noise, and indirect effects associated with the spaceflight environment such as high workload, isolation, and confinement (Kanas and Manzey, 2008; Cucinotta et al., 2014; Law et al., 2014). These factors also contribute to various symptoms often observed during space missions. For example, astronauts suffer from space motion sickness (SMS) (Lackner and DiZio, 2006) and decreased sleep duration (Barger et al., 2014). Clinically significant neuro-ophthalmological symptoms (e.g., globe flattening, decreased visual acuity, and increased intracranial pressure) have also been reported in long-duration missions, coined under the term Spaceflight-Associated Neuro-Ocular Syndrome (SANS) (Paez et al., 2020). Numerous forms of spaceflight-related structural brain changes are proven, caused by the lack of hydrostatic pressure under microgravity, including upward shift of the brain, upward cerebrospinal fluid shift, ventricular volume gray matter volume decline (for a detailed review see Stahn and Kühn, 2021; Roy-O’Reilly et al., 2021).

In contrast to clear neuroimaging findings, results regarding cognitive performance are far from conclusive (Strangman et al., 2014). Although numerous studies have examined cognitive functions in space, the reason behind the observed changes is not yet clear. One potential source of cognitive changes is the microgravity-induced fluid shift to the upper body (Thornton et al., 1987) and an upward shift of the brain (Roberts et al., 2017). Fortunately, these changes can also be investigated under terrestrial conditions. The 6° head-down tilt bedrest (HDBR), for example, is a well-established ground-based analog for spaceflight-induced physiological alterations, including cardiovascular changes (Pavy Le-Traon et al., 2007; Barbic et al., 2019), cephalic fluid shift (Hargens and Vico, 2016), upward and posterior brain shifts, increased density of brain tissue at the vertex, increased ventricular volume (Roberts et al., 2015; Roy-O’Reilly et al., 2021), and alterations in brain connectivity (Cassady et al., 2016). HDBR also enables testing the effectiveness of countermeasures mitigating spaceflight associated performance declines, such as artificial gravity (AG) and exercise (Tays et al., 2021). However, while HDBR is thought to simulate microgravity-induced physiologic changes (Pavy Le-Traon et al., 2007), it is still debatable whether HDBR is a reliable model for space-induced cognitive deterioration. Therefore, this review aims at comparing the effects of spaceflight and HDBR on cognitive functions.

In the following, the results of relevant studies will be summarized using seven main categories according to the cognitive function most required for the given task. The categories are as follows: Sensorimotor skills, Time estimation, Attention and psychomotor speed, Memory, Executive functions, Reasoning, and mathematical processing, and Cognitive processing of emotional stimuli. Note that more complex tasks involve several cognitive domains and which of these is primary is not always clear. Results regarding behavioral performance, related brain electrical activity and brain imaging results are discussed under each category. Readaptation of cognitive functions after spaceflight/HDBR exposure are also summarized by comparing pre- and post-measurement points in relevant studies in each category. Relevant information (e.g., results, duration, number of subjects, the presence or absence of in-flight/in-HDBR measurements) of all space and HDBR studies are also summarized for each category under Table 1, Table 2, Table 3, Table 4, Table 5, Table 6, Table 7.

**TABLE 1:** Changes in sensorimotor skills, based on the results of the reviewed space and HDBR studies. Results regarding behavioral performance are summarized task wise. Results are presented for pre versus in-flight/in-HDBR comparisons or during in-flight/in-HDBR as Changes IN space/HDBR, while pre versus post-flight/post-HDBR comparisons are presented as Changes AFTER space/HDBR. Number of subjects, duration of in-flight/in-HDBR are also presented. The presence or absence of pre/in/post measures and control studies (ctrl) are marked with Y (yes), N (no) in each case. Please note that follow-up measures are not included in this table. Studies based on spaceflight missions are illustrated with light-gray background. Studies based on HDBR exposure are illustrated with white background. Additional notes: ↑ (=performance improvement); ↓ (=performance deterioration); ↔ (=unchanged performance); n.m. (=no measurement); n.a. (=not analyzed). For consistency in the presentation of results, the uncorrected findings are reported from the Koppelmans et al. (2015) study.

Cognitive test	Changes IN space/HDBR	Changes AFTER space/HDBR	Subjects	Duration	Pre	In	Post	Ctrl	Authors
**Tracking tasks**									
Unstable tracking	↓	↔	1	8 days	Y	Y	Y	N	Manzey et al. (1993),
									Manzey et al. (1995)*
	↓	↓	1	438 days	Y	Y	Y	N	Manzey et al. (1998)
	↓	↓	1	20 days	Y	Y	Y	N	Manzey et al. (2000)
	↔/↑**	n.a	4	16 days	Y	Y	Y	N	Eddy et al. (1998)
	↓	↔	3	half year	Y	Y	Y	N	Bock et al. (2010)
	n.m	↔	8	half year	Y	N	Y	Y	Moore et al. (2019)
	↔	↔	5	half year	Y	Y	Y	N	Petit et al. (2019)
	↔	↔	8	16 days	Y	Y	Y	N	Shehab et al. (1998)
Fittsberg	↓	↔	4	8 days	Y	Y	Y	Y	Newman and Lathan (1999)
Pursuit tracking	↔	↔	4–6	16 days	Y	Y	Y	N	Bock et al. (2001),
									Fowler et al. (2000)*
	n.m	↔	14	half year	Y	N	Y	N	Kornilova et al. (2016)
	↑	↔	18	30 days	Y	Y	Y	N	DeRoshia and Greenleaf (1993)
**Pointing arm movement**	↓	↔	4	16 days	Y	Y	Y	N	Bock et al. (2001)
	↓	↔	3	10–172 days	Y	Y	Y	N	Berger et al. (1997)
**Motor praxis**	↔	↓	1	1 year	Y	Y	Y	Y	Garrett-Bakelman et al. (2019)
	↓	↔	8	60 days	Y	Y	Y	N	Basner et al. (2021)
**Purdue/Grooved pegboard**	n.m	↓	15	half year	Y	N	Y	N	Tays et al. (2021)
	n.m	↓	8	half year	Y	N	Y	Y	Moore et al. (2019)
	n.m	↓	13	half year	Y	N	Y	N	Mulavara et al. (2018)
	n.m	↔	19	70 days	Y	N	Y	N	Mulavara et al. (2018)
	↑	↑	10	70 days	Y	Y	Y	Y	Koppelmans et al. (2015)
	↔	n.a	8	60 days	Y	Y	Y	N	Tays et al. (2022)
**Driving simulation**	n.m		8	half year	Y	N	Y	Y	Moore et al. (2019)
Mountain road		↓							
Cone course		↔							

*The results of the same study are reported in several articles.

**Different results belong to different subjects.

**TABLE 2:** Changes in time estimation, based on the results of the reviewed space and HDBR studies. Results regarding behavioral performance are summarized task wise. Results are presented for pre versus in-flight/in-HDBR comparisons or during in-flight/in-HDBR as Changes IN space/HDBR, while pre versus post-flight/post-HDBR comparisons are presented as Changes AFTER space/HDBR. Number of subjects, duration of in-flight/in-HDBR are also presented. The presence or absence of pre/in/post measures and control studies (ctrl) are marked with Y (yes), N (no) in each case. Please note that follow-up measures are not included in this table. Studies based on spaceflight missions are illustrated with light-gray background. Studies based on HDBR exposure are illustrated with white background. Additional notes: ↑ (=performance improvement); ↓ (=performance deterioration); ↔ (=unchanged performance); n.m. (=no measurement); n.a. (=not analyzed).

Cognitive test	Changes IN space/HDBR	Changes AFTER space/HDBR	Subjects	Duration	Pre	In	Post	Ctrl	Authors
**Time estimation**	↔	↔	1	6 days	Y	Y	Y	N	Benke et al. (1993a)
**Regularity of timing**	↓	↓	3	17–180 days	Y	Y	Y	Y	Semjen et al. (1998)
**Differential Reinforcement**	↔	↔	4	10 days	Y	Y	Y	N	Kelly et al. (2005)
**Temporal Bisection**	↓	↓	17	15 days	Y	Y	Y	N	Qian et al. (2021)

**TABLE 3:** Changes in attention and psychomotor speed, based on the results of the reviewed space and HDBR studies. Results regarding behavioral performance and task related brain electrical activity are summarized task wise. Results are presented for pre versus in-flight/in-HDBR comparisons or during in-flight/in-HDBR as Changes IN space/HDBR, while pre versus post-flight/post-HDBR comparisons are presented as Changes AFTER space/HDBR. Number of subjects, duration of in-flight/in-HDBR are also presented. The presence or absence of pre/in/post measures and control studies (ctrl) are marked with Y (yes), N (no) in each case. Please note that follow-up measures are not included in this table. Studies based on spaceflight missions are illustrated with light-gray background. Studies based on HDBR exposure are illustrated with white background. Additional notes: ↑ (=performance improvement); ↓ (=performance deterioration); ↔ (=unchanged performance); n.m. (=no measurement); n.a. (=not analyzed); DTC (=dual task cost: the difference between the performance of dual and single tasks). For consistency in the presentation of results, the uncorrected findings are reported from the Koppelmans et al. (2015) study.

Cognitive test	Changes IN space/HDBR	Changes AFTER space/HDBR	Subjects	Duration	Pre	In	Post	Ctrl	Authors
**Simple RT**	↓	↔	4–6	16 days	Y	Y	Y	N	Fowler et al. (2000)
	↔	↔	1	6 days	Y	Y	Y	N	Benke et al. (1993b)
									Benke et al. (1993a)*
	n.m	↔	8	half year	Y	N	Y	Y	Moore et al. (2019)
	↔	↓	24	60 days	Y	Y	Y	Y	Lipnicki et al. (2009a)
	↔	↔	18	30 days	Y	Y	Y	N	DeRoshia and Greenleaf (1993)
**Choice/Complex RT**	↔	↔	1	6 days	Y	Y	Y	N	Benke et al. (1993b)
									Benke et al. (1993a)*
	↔	↔	3	half year	Y	Y	Y	N	Bock et al. (2010)
	↔	↔	13	70 days	Y	Y	Y	N	Mahadevan et al. (2021)
**PVT**	↔	↔	5	10–16 days	Y	Y	Y	N	Dijk et al. (2001)
	↔	↔	24	half year	Y	Y	Y	N	Jones et al. (2022)
	↔	↓	1	1 year	Y	Y	Y	Y	Garrett-Bakelman et al. (2019)
	↔	↔	8	60 days	Y	Y	Y	N	Basner et al. (2021)
**DSST**	↔	↑	4	10 days	Y	Y	Y	N	Kelly et al. (2005)
	n.m	↔	15	half year	Y	N	Y	N	Tays et al. (2021)
	↔	↓	1	1 year	Y	Y	Y	Y	Garrett-Bakelman et al. (2019)
	↔	↑	10–18	70 days	Y	Y	Y	Y/N	Koppelmans et al. (2015),
									Lee et al. (2019)*
	↔	n.a	8	60 days	Y	Y	Y	N	Tays et al. (2022)
	↔	↔	8	60 days	Y	Y	Y	N	Basner et al. (2021)
	↔	n.a	6	28 days	Y	Y	Y	Y	Pavy Le-Traon et al. (1994)
	↑	↔	18	30 days	Y	Y	Y	N	DeRoshia and Greenleaf (1993)
**Squares**	↑	n.a	6	28 days	Y	Y	Y	N	Pavy Le-Traon et al. (1994)
**Counting oddball**	↔	↔	13	70 days	Y	Y	Y	N	Mahadevan et al. (2021)
**3-stimulus-oddball**			20	60 days	Y	Y	Y	N	Brauns et al. (2021a)
Performance	↓	↓							
P3a amplitude	↓	↓							
P3b amplitude	↓	↓							
**Tapping**			18	30 days	Y	Y	Y	N	DeRoshia and Greenleaf (1993)
Two fingers	↔	↔							
Preferred hand	↑	↔							
Non-preferred hand	↑	↔							
**Pattern comparison**	↔	n.a	6	28 days	Y	Y	Y	Y	Pavy Le-Traon et al. (1994)
	↑	↔	18	30 days	Y	Y	Y	N	DeRoshia and Greenleaf (1993)
**Dual-task**									
Tracking	↓	↔	1	8 days	Y	Y	Y	N	Manzey et al. (1995)
Sternberg	↓	↔							
Tracking	↔	↔	1	438 days	Y	Y	Y	N	Manzey et al. (1998)
Sternberg	↓	↑							
Tracking	↔	n.a	4	16 days	Y	Y	Y	N	Eddy et al. (1998)
Tracking	↔	↔	8	16 days	Y	Y	Y	N	Shehab et al. (1998)
Sternberg	↔	↔							
Tracking	↔	↔	4–6	16 days	Y	Y	Y	N	Fowler et al. (2000),
RT task	↔	↔							Bock et al. (2001)*
Tracking DTC	↓/↔***	n.a	3	half year	Y	Y	Y	N	Bock et al. (2010)
RT task DTC	↔	n.a							
Tracking	n.m	↓	8	half year	Y	N	Y	Y	Moore et al. (2019)
Code entering	n.m	↔							
RT task DTC	↔	↔	15	half year	Y	Y	Y	N	Tays et al. (2021)
Counting oddball DTC	↔	↔							
RT task DTC	↔	n.a	8	60 days	Y	Y	Y	N	Tays et al. (2022)
Counting oddball DTC	↔	n.a							
RT task	↔	↔	13–18	70 days	Y	Y	Y	Y/N	Lee et al. (2019),
Counting oddball	↔	↔							Mahadevan et al. (2021),
RT task DTC	↔	↔							Yuan et al. (2016)*
Counting oddball DTC	↔	↔							
**Directed attention**									
Random switching task	↓/↔**	↓/↔**	4	16 days	Y	Y	Y	N	Eddy et al. (1998)
	↔	↔	8	16 days	Y	Y	Y	N	Shehab et al. (1998)

*The results of the same study are reported in several articles.

**Different results belong to different subjects.

***Different results belong to different type of dual-task.

**TABLE 4:** Changes in memory, based on the results of the reviewed space and HDBR studies. Results regarding behavioral performance and task related brain electrical activity are summarized task wise. Results are presented for pre versus in-flight/in-HDBR comparisons or during in-flight/in-HDBR as Changes IN space/HDBR, while pre versus post-flight/post-HDBR comparisons are presented as Changes AFTER space/HDBR. Number of subjects, duration of in-flight/in-HDBR are also presented. The presence or absence of pre/in/post measures and control studies (ctrl) are marked with Y (yes), N (no) in each case. Please note that follow-up measures are not included in this table. Studies based on spaceflight missions are illustrated with light-gray background. Studies based on HDBR exposure are illustrated with white background. Additional notes: ↑ (=performance improvement); ↓ (=performance deterioration); ↔ (=unchanged performance); n.m. (=no measurement); n.a. (=not analyzed). For consistency in the presentation of results, the uncorrected findings are reported from the Koppelmans et al. (2015) study.

Cognitive test	Changes IN space/HDBR	Changes AFTER space/HDBR	Subjects	Duration	Pre	In	Post	Ctrl	Authors
**Short-term and working memory**									
Sternberg	↔	↔	1	8 days	Y	Y	Y	N	Manzey et al. (1993),
									Manzey et al. (1995)*
	↔	↓	1	438 days	Y	Y	Y	N	Manzey et al. (1998)
	↔	n.a	4	16 days	Y	Y	Y	N	Eddy et al. (1998)
	↔	↔	4	8 days	Y	Y	Y	Y	Newman and Lathan (1999)
	↓	↔	4	10 days	Y	Y	Y	N	Kelly et al. (2005)
	↔	↔	8	16 days	Y	Y	Y	N	Shehab et al. (1998)
	↑	n.a	6	28 days	Y	Y	Y	Y	Pavy Le-Traon et al. (1994)
		↔	↔	18	30 days	Y	Y	Y	N	DeRoshia and Greenleaf (1993)
Continuous recognition memory	↔	n.a	4	16 days	Y	Y	Y	N	Eddy et al. (1998)
	↔	↔	8	16 days	Y	Y	Y	N	Shehab et al. (1998)
	↑	n.m	22	60 days	Y	Y	Y	N	Friedl-Werner et al. (2020)
Running memory continuous performance	↔	↔	13	60–90 days	Y	Y	Y	N	Seaton et al. (2009)
**Probed recall memory**	↓	↔	5	10–16 days	Y	Y	Y	N	Dijk et al. (2001)
**Visual object learning**	↔	↓	1	1 year	Y	Y	Y	Y	Garrett-Bakelman et al. (2019)
	↔	↔	8	60 days	Y	Y	Y	N	Basner et al. (2021)
**Code memory delayed**	↔	↔	13	60–90 days	Y	Y	Y	N	Seaton et al. (2009)
**Spatial working memory**									
Line orientation memory	↔	↔	5	half year	Y	Y	Y	Y	McIntyre et al. (2001)
	↔	↔	1	6 days	Y	Y	Y	N	Benke et al. (1993b),
									Benke et al. (1993a)*
Performance	↓	↓	5	half year	Y	Y	Y	N	Takács et al. (2021)
P3a amplitude	↓	↓							
P3b amplitude	↓	↓							
Array match to sample	n.m	↔	8	half year	Y	N	Y	Y	Moore et al. (2019)
Grid match to sample	↔	↔	13	60–90 days	Y	Y	Y	N	Seaton et al. (2009)
Spatial location	↔	↔	1	6 days	Y	Y	Y	N	Benke et al. (1993b),
									Benke et al. (1993a)*
Manikin	↔	n.a	4	16 days	Y	Y	Y	N	Eddy et al. (1998)
	↔	↔	8	16 days	Y	Y	Y	N	Shehab et al. (1998)
	↑	n.a	6	28 days	Y	Y	Y	Y	Pavy Le-Traon et al. (1994)
	↑	↔	18	30 days	Y	Y	Y	N	DeRoshia and Greenleaf (1993)
Spatial matrix rotation	↓/↔**	↓/n.a.**	4	16 days	Y	Y	Y	N	Eddy et al. (1998)
	↔	↔	8	16 days	Y	Y	Y	N	Shehab et al. (1998)
Mental rotation	↑	↑	8	14–199 days	Y	Y	Y	Y	Leone et al. (1995)
	↑	↑	3	1–6 months	Y	Y	Y	Y	Matsakis et al. (1993)
Spatial working memory task	n.m	↔	15	half year	Y	Y	Y	N	Tays et al. (2021)
	↔	n.a	8	60 days	Y	Y	Y	N	Tays et al. (2022)
	↔	n.a	18	70 days	Y	Y	Y	N	Lee et al. (2019)
Thurstone’s 2D card rotation	n.m	↔	15	half year	Y	Y	Y	N	Tays et al. (2021)
	↔	n.a	8	60 days	Y	Y	Y	N	Tays et al. (2022)
	↑	↑	10–18	70 days	Y	Y	Y	Y/N	Koppelmans et al. (2015),
									Lee et al. (2019),
									Cassady et al. (2016)*
3D cube mental rotation	↔	↔	15	half year	Y	Y	Y	N	Tays et al. (2021)
	↔	n.a	8	60 days	Y	Y	Y	N	Tays et al. (2022)
	↑	↑	10–18	70 days	Y	Y	Y	Y/N	Koppelmans et al. (2015)
									Lee et al. (2019)
									Cassady et al. (2016)*
**Recognition faces**									
Learned-on-ground face	↔	n.a	3	15 days-6 months	Y	Y	Y	N	de Schonen et al. (1998)
Learned-in-flight face	↓	n.m							
**Response sequences**	↑	↑	4	10 days	Y	Y	Y	N	Kelly et al. (2005)

*The results of the same study are reported in several articles.

**Different results belong to different subjects.

**TABLE 5:** Changes in executive functions, based on the results of the reviewed space and HDBR studies. Results regarding behavioral performance are summarized task wise. Results are presented for pre versus in-flight/in-HDBR comparisons or during in-flight/in-HDBR as Changes IN space/HDBR, while pre versus post-flight/post-HDBR comparisons are presented as Changes AFTER space/HDBR. Number of subjects, duration of in-flight/in-HDBR are also presented. The presence or absence of pre/in/post measures and control studies (ctrl) are marked with Y (yes), N (no) in each case. Please note that follow-up measures are not included in this table. Studies based on spaceflight missions are illustrated with light-gray background. Studies based on HDBR exposure are illustrated with white background. Additional notes: ↑ (=performance improvement); ↓ (=performance deterioration); ↔ (=unchanged performance); n.m. (=no measurement); n.a. (=not analyzed).

Cognitive test	Changes IN space/HDBR	Changes AFTER space/HDBR	Subjects	Duration	Pre	In	Post	Ctrl	Authors
**Stroop**	↔	↔	1	6 days	Y	Y	Y	N	Benke et al. (1993b)
	↔	↔	3	11 days	Y	Y	Y	Y	Pattyn et al. (2009),
									Pattyn et al. (2005)*
**Abstract matching**	↔	↓	1	1 year	Y	Y	Y	Y	Garrett-Bakelman et al. (2019)
	↔	↔	8	60 days	Y	Y	Y	N	Basner et al. (2021)
**2-back**	↔	↓	1	1 year	Y	Y	Y	Y	Garrett-Bakelman et al. (2019)
	↔	↔	8	60 days	Y	Y	Y	N	Basner et al. (2021)
	↑	↑	22	60 days	Y	Y	Y	Y	Lipnicki et al. (2009a)
**BART**	↔	↓	1	1 year	Y	Y	Y	Y	Garrett-Bakelman et al. (2019)
	↔	↔	8	60 days	Y	Y	Y	N	Basner et al. (2021)
	↔	↔	16	45 days	Y	Y	Y	N	Rao et al. (2014)
**Flanker**	↑	↑	19	60 days	Y	Y	Y	Y	Lipnicki et al. (2009a)
**Iowa Gambling**			20–25	60 days					Lipnicki et al. (2009a),
									Lipnicki et al. (2009b)*
1	↔	n.m.			Y	Y	N	N	
2	n.m.	↑**			N	Y	Y	N	

*The results of the same study are reported in several articles.

**Post versus in-HDBR comparison.

**TABLE 6:** Changes in reasoning and mathematical processing, based on the results of the reviewed space and HDBR studies. Results regarding behavioral performance are summarized task wise. Results are presented for pre versus in-flight/in-HDBR comparisons or during in-flight/in-HDBR as Changes IN space/HDBR, while pre versus post-flight/post-HDBR comparisons are presented as Changes AFTER space/HDBR. Number of subjects, duration of in-flight/in-HDBR are also presented. The presence or absence of pre/in/post measures and control studies (ctrl) are marked with Y (yes), N (no) in each case. Please note that follow-up measures are not included in this table. Studies based on spaceflight missions are illustrated with light-gray background. Studies based on HDBR exposure are illustrated with white background. Additional notes: ↑ (=performance improvement); ↓ (=performance deterioration); ↔ (=unchanged performance); n.m. (=no measurement); n.a. (=not analyzed).

Cognitive test	Changes IN space/HDBR	Changes AFTER space/HDBR	Subjects	Duration	Pre	In	Post	Ctrl	Authors
**Reasoning**									
Grammatical	↔	↑	1	8 days	Y	Y	Y	N	Manzey et al. (1993)
	↔	↔	1	438 days	Y	Y	Y	N	Manzey et al. (1998)
	↑	n.a	6	28 days	Y	Y	Y	Y	Pavy Le-Traon et al. (1994)
	↑	↔	18	30 days	Y	Y	Y	N	DeRoshia and Greenleaf (1993)
Matrix	↔	↓	1	1 year	Y	Y	Y	Y	Garrett-Bakelman et al. (2019)
	↔	↔	8	60 days	Y	Y	Y	N	Basner et al. (2021)
**Mathematical processing**	↓/↔**	↓/n.a**	4	16 days	Y	Y	Y	N	Eddy et al. (1998)
	↔	↔	8	16 days	Y	Y	Y	N	Shehab et al. (1998)
	↔	↔	13	60–90 days	Y	Y	Y	N	Seaton et al. (2009)

**Different results belong to different subjects.

**TABLE 7:** Changes in cognitive processing of emotional stimuli, based on the results of the reviewed space and HDBR studies. Results regarding behavioral performance are summarized task wise. Results are presented for pre versus in-flight/in-HDBR comparisons or during in-flight/in-HDBR as Changes IN space/HDBR, while pre versus post-flight/post-HDBR comparisons are presented as Changes AFTER space/HDBR. Number of subjects, duration of in-flight/in-HDBR are also presented. The presence or absence of pre/in/post measures and control studies (ctrl) are marked with Y (yes), N (no) in each case. Please note that follow-up measures are not included in this table. Studies based on spaceflight missions are illustrated with light-gray background. Studies based on HDBR exposure are illustrated with white background. Additional notes: ↑ (=performance improvement); ↓ (=performance deterioration); ↔ (=unchanged performance); n.m. (=no measurement); n.a. (=not analyzed).

Cognitive test	Changes IN space/HDBR	Changes AFTER space/HDBR	Subjects	Duration	Pre	In	Post	Ctrl	Authors
**Emotional Stroop**	↓	↔	3	11 days	Y	Y	Y	Y	Pattyn et al. (2009)
									Pattyn et al. (2005)*
**Facial emotion recognition**	↔	↓	1	1 year	Y	Y	Y	Y	Garrett-Bakelman et al. (2019)
	↓	↓	8	60 days	Y	Y	Y	N	Basner et al. (2021)
**Affective picture evaluation**	n.m	n.m	10	30 days	N	N	Y	Y	Brauns et al. (2019)
**Emotional flanker**	↓	↔	15	45 days	Y	Y	Y	N	Liu et al. (2012)
**Dot-probe**	↓	↔	17	15 days	Y	Y	Y	N	Jiang et al. (2022)

*The results of the same study are reported in several articles.

## Cognitive results of space and head-down tilt bedrest studies in each category

Relevant space and HDBR studies published before 30 June 2022 were detected by searching Google Scholar and ResearchGate using the following terms: bedrest; head-down tilt; HDBR; weightlessness; microgravity; space; space mission; cognitive; psychomotor; reaction time; EEG; fMRI. Additional articles were identified from reference lists. As performance deterioration of even a single astronaut can pose a hazard to mission success, individual factors causing susceptibility to space-related stress factors should also be considered. Therefore, even though findings of case and low sample size studies may be less generalizable for the overall population, sample size was not an exclusion criterion in this review. Additionally, while HDBR is important for testing exercise and AG as countermeasures against space-induced physiological changes, cognitive results for HDBR are presented in this review regardless of the presence or absence of exercise during HDBR, while for HDBR studies investigating AG protocol(s) as a countermeasure, results are only discussed for the HDBR group without AG (Basner et al., 2021; Tays et al., 2022). Significant between-group differences are also noted in the text.

Studies and tasks were excluded if the spaceflight/bedrest duration was less than 6 days (Basner et al., 2018 for example), or if the main focus was on balance, locomotion, gross body movement, vestibular system, and spatial orientation. In each category, results are presented based on the administered tasks. Please note that studies applying several cognitive measures are listed under each category/task. The review also includes articles describing different aspects of the same research (such as a) Fowler et al., 2000; Bock et al., 2001; b) Lee et al., 2019; Mahadevan et al., 2021; Yuan et al., 2016; Koppelmans et al., 2015; Cassady et al., 2016; c) Benke et al., 1993a; Benke et al., 1993b; d) Pattyn et al., 2005; Pattyn et al., 2009; e) Manzey et al., 1993; Manzey et al., 1995). Results of short and long spaceflight and HDBR are discussed separately. Space missions with an average length above half a year are considered as long duration. Additionally, the term astronaut is used for space travelers throughout the text for the sake of consistency.

### Sensorimotor skills

This section summarizes the results of the studies tapping into sensorimotor skills (results are further detailed in Table 1). To evaluate spaceflight and HDBR-related changes in eye-hand coordination, numerous studies used certain types of the Tracking task, including the Unstable tracking task, the Fittsberg task, and the Pursuit tracking task. In the Unstable tracking task, subjects have to keep an unstable target in the center of a marked target area (Manzey et al., 1993; 1995; 1998; 2000; Eddy et al., 1998; Shehab et al., 1998; Bock et al., 2010), or maintain a target inside an unstable object (Moore et al., 2019). Most studies using the Unstable tracking task found significantly worse in-flight performance during both short-duration and long duration space missions. More errors were detected during an 8-days-long (Manzey et al., 1993, 1995), and a 3-weeks-long short duration spaceflight (Manzey et al., 2000). As for long-duration space missions, in-flight performance deterioration was found during the first 2–3 weeks of a 438-days-long space flight (Manzey et al., 1998) or even persisted over several months in-flight (Bock et al., 2010). The Docking task reported by Petit et al. (2019), which was a Soyuz vehicle docking simulation task under two scenarios, could also be considered an Unstable tracking task version. The team reported uncompromised docking performance during a half-year-long mission with an increased number of slower than median RTs (i.e., time to achieve successful docking within a timeframe). In contrast to these results, intact performance was found during a 16-days-long mission (Eddy et al., 1998). Furthermore, Eddy et al. (1998) even reported improved performance in 1 out of the 4 astronauts, but contrary to other tracking task studies, they fitted a learning curve to the pre-flight measurement points and used a different performance index. An additional long-duration space study only compared pre-flight and post-flight measurement points of a half-year-long space mission and found no deterioration in the Unstable tracking task (Moore et al., 2019). Tracking performance was also investigated by the Fittsberg task, and the Pursuit tracking task. The Fittsberg task is a combination of a short-term memory (Sternberg) task and a motor control test. In this task, subjects must decide which of the displayed letters is part of the memory set and then manually navigate the cursor from the initial center position to the target letter. Here we only discuss results regarding movement time as an index for sensorimotor performance, while decision time reflects short term memory and therefore are detailed under the Memory section. Increased in-flight movement time was detected during an 8-days-long space mission in the Fittsberg task (Newman and Lathan, 1999). The Pursuit tracking task requires participants to track a moving target with or without seeing their hand. Intact performance was found in this task during a 16-days-long space mission (Fowler et al., 2000; Bock et al., 2001), however, during space flight, tracking movement started to follow an ellipsoid response path instead of a circular one, which became more closely aligned with the long body axis of the subjects. This alteration also persisted 9 days after post-flight with a trend toward normalization (Bock et al., 2001). An additional space study with 14 astronauts applied a version of the Pursuit tracking task (Kornilova et al., 2016). They measured both manual and visual tracking performance in a task where subjects had to visually follow a moving target (linear and sinusoidal) and manually replicate the movement by manually controlling the movement of a second object displaced from the target. Measurements before and after half-year-long space missions revealed that even though visual tracking worsened in the first few days after return, sensorimotor performance remained intact in this task.

Regarding readaptation of tracking performance to terrestrial conditions, the majority of the studies reporting worse performance during in-flight did not find sensorimotor performance deterioration within a week after landing. The 2 exceptions were case studies about a 20-day-long space mission (Manzey et al., 2000), and a 438-day-long space mission (Manzey et al., 1998). Regardless of mission length, both studies found performance decrement to be limited to the first 1–2 weeks of post-flight, while later post-flight sessions (on days 72–73 for Manzey et al., 2000; and on day 168 for Manzey et al., 1998) showed successful readaptation in the Tracking task.

As for HDBR, only two 16-days-long HDBR studies measured tracking performance, one of them reported no decrement between pre- and in-HDBR during the Unstable tracking task (Shehab et al., 1998), while the other study even found improved performance during a version of the Pursuit tracking task in which subjects had to track and hit a moving target object (DeRoshia and Greenleaf, 1993).

Sensorimotor skills were also investigated by the Pointing arm movement task by two space studies (Berger et al., 1997; Bock et al., 2001), while HDBR investigations are not yet available. In this task, participants are instructed to point at visual targets with (Berger et al., 1997) or without seeing their hands (Bock et al., 2001). These studies investigated performance of 3-4 astronauts during short- and long-duration space missions and reported increased in-flight movement time and no pre- to post-flight differences.

Sensorimotor speed was assessed by a one-year-long space study (Garrett-Bakelman et al., 2019) and by a 60-days-long HDBR study (Basner et al., 2021) with the Motor praxis task. In this task, participants are instructed to click on consecutive squares that appear randomly on the screen and become smaller with each appearance. While results showed no in-flight performance decrease during the one-year-long mission of Garrett-Bakelman et al. (2019), they did report decreased post-flight performance. As for HDBR, Basner et al. (2021) reported decreased in-HDBR sensorimotor speed in the Motor praxis task, but found no pre- to post-HDBR differences.

Manual dexterity and fine motor control was investigated in 3 long-duration space studies (Mulavara et al., 2018; Moore et al., 2019; Tays et al., 2021), and in 3 60–70 days long HDBR studies (Koppelmans et al., 2015; Mulavara et al., 2018; Tays et al., 2022). Please note that Mulavara et al. (2018) investigated these skills under both long-duration spaceflight and HDBR. These studies either used the Purdue pegboard test (Koppelmans et al., 2015; Moore et al., 2019; Tays et al., 2021; Tays et al., 2022) or the Grooved pegboard test (Mulavara et al., 2018). In both versions of the pegboard test, subjects are instructed to place pegs into holes as quickly as possible, however, in the Grooved pegboard test, pegs must be rotated to match the hole before they can be inserted. The available space studies only evaluated pre- and postflight differences after half-year-long space missions. Using the data of 8 astronauts, Moore et al. (2019) found a significant drop in performance in the Purdue pegboard test with the non-dominant hand, and a trend towards deterioration with the dominant hand and bimanual task execution directly after returning to Earth, while performance reached pre-flight level 4 days after landing. Additionally, they found no such time effects in a ground control group of 12 subjects. In their study with 15 astronauts, Tays et al. (2021) also found significant performance decrement in bimanual motor coordination in the same task during their earliest post-flight measurement (4 days after landing), while reporting complete readaptation of performance in their later post-flight measurement (after 1 month of landing). Using the Grooved pegboard test in their study with 13 astronauts, Mulavara et al. (2018) also found significant performance deterioration with the dominant hand 1 day after returning from a half-year-long space mission. Contrary, Mulavara et al. (2018) reported unchanged performance directly after 70 days of HDBR with 19 subjects. The other two HDBR studies used the bimanual version of the Purdue pegboard test (70 days of HDBR, Koppelmans et al., 2015; 60 days of HDBR, Tays et al., 2022). Tays et al. (2022) compared pre- and in-HDBR measurement points and found intact sensorimotor performance, while comparing pre-HDBR with in-HDBR and post-HDBR, Koppelmans et al. (2015) even found improved sensorimotor performance on the last day in-HDBR and on day 8 and 12 of post-HDBR.

Finally, Moore et al. (2019) also assessed two complex sensorimotor driving simulation tasks along with the above reported Unstable tracking task before and after a half-year-long space mission. In the Mountain road simulation part of this task, subjects are instructed to drive a car as fast as possible on a mountain road while maintaining their position in the right lane. In the Cone course driving task, subjects must slalom around the cones with the car as quickly as possible. While no pre- to post-flight difference was found regarding the Unstable tracking task and the Cone driving simulation task, Moore et al. (2019) did report malaise directly after landing in the Mountain driving simulation task which did recover to baseline 4 days after landing. To our knowledge, no similar tasks were yet assessed under HDBR.

### Time estimation

The potential effect of spaceflight on time estimation was investigated in 3 space studies (Benke et al., 1993a; Semjen et al., 1998; Kelly et al., 2005) (see Table 2). Estimation of the length of various time periods was assessed in a case study of a 6-day-long space mission (Benke et al., 1993a). A tendency to underestimate longer periods (6, 8 and 10 s) was reported. In the Semjen et al. (1998) study with 3 astronauts, the task was to synchronize tapping to a metronome and to maintain tempo after the metronome stopped. The astronauts kept taping faster than requested in space as well as post-flight compared to pre-flight, while no such changes were observed in control subjects. The 4 astronauts included in the experiment of Kelly et al. (2005) earned points for each keystroke separated by 12 s or more, a task required to maintain a slow tapping rate. The astronauts performed equally well during the 10 days of in-flight as well as on Earth.

In the only available 15-day-long HDBR investigation (Qian et al., 2021), participants had to decide if the length of a stimulus was closer to the low (300 ms) or the high (900 ms) end of the range. Stimuli were pictures with emotional content (neutral, fear or disgust). Results indicated a diminished in-HDBR and post-HDBR time sensitivity (variability of estimation) relative to baseline and overestimation of fear stimuli in the middle phase of HDBR.

### Attention and psychomotor speed

Spaceflight and HDBR-related changes in psychomotor speed and attention, divided attention and directed attention were investigated in several studies (see Table 3). Regarding psychomotor speed and attention, the following tasks were administered in the available space literature: Simple reaction time (RT) tasks, Choice/Complex RT tasks, Psychomotor vigilance task (PVT), and the Digit-symbol substitution task (DSST). Simple RT task requires one specific response (e.g., a button press) whenever any stimulus appears (e.g., circular icon) or changes on the screen (Benke et al., 1993a; 1993b; Fowler et al., 2000; Moore et al., 2019). Choice/Complex RT tasks include various stimuli (e.g., various symbols, various stimulus positions) and each type of stimuli requires different responses (Benke et al., 1993a; 1993b; Bock et al., 2010). Besides two classical Choice/Complex RT tasks, Bock et al. (2010) also included two additional task variations, one in which the response button corresponded to a clockwise 90-degree rotation of the stimulus, and another one in which the response button had to be pressed four times in a pre-established rhythm. PVT is also a reaction time task, in which subjects are instructed to press a button as quickly as possible when a millisecond counter appears in the box on the screen and subjects receive feedback regarding their RT after each response (Dijk et al., 2001; Garrett-Bakelman et al., 2019; Jones et al., 2022). Most space studies found intact performance during both short- and long-duration space missions in the Simple RT, the Choice/Complex RT, and the PVT tasks (Benke et al., 1993a; 1993b; Dijk et al., 2001; Bock et al., 2010; Garrett-Bakelman et al., 2019; Jones et al., 2022). Contrary to most studies, Fowler et al. (2000) found increased error rates in the Simple RT task at the end of a 2-weeks-long space mission. Although another short-duration space study reported decreased in-flight performance (increased number of lapses) in the PVT task (Dijk et al., 2001), they obtained only a marginally significant main effect and did not describe pre-flight/in-flight/post-flight pairwise comparisons. Concerning in-flight/post-flight performance differences, no signs of performance deterioration was found after returning to Earth in the majority of these studies. The only exception was the study of Garrett-Bakelman et al. (2019), which reported decreased performance in a short version of the PVT task after a one-year-long mission. This task was also assessed in a large-scale study involving 24 astronauts on 6 months long ISS missions (Jones et al., 2022). Their main finding was a negative association between performance and the amount of sleep. Although astronauts slept less in space, compared to pre-flight levels speed was not compromised either during in-flight or post-flight, perhaps due to the fact that performance was by far the worst in the week before launch compared to any other sessions. Another task for measuring psychomotor speed and attention is the DSST, which was assessed in three space studies (Kelly et al., 2005; Garrett-Bakelman et al., 2019; Tays et al., 2021). In the most common version of this task, subjects are instructed to match numbers and symbols according to a given key of digit-symbol pairs. Alternatively, in the study of Kelly et al. (2005), astronauts had to match numbers and arrays of black and white boxes instead of symbols. Regarding spaceflight-related changes, one short and two long durational space studies were conducted using DSST (Kelly et al., 2005; Garrett-Bakelman et al., 2019; Tays et al., 2021). No deterioration was found during or after a 10-days-long space mission (Kelly et al., 2005), or after returning from a half-year-long space mission (Tays et al., 2021). Only Garrett-Bakelman et al. (2019) found decreased performance in their case study after a one-year-long flight.

HDBR studies showed a similar pattern, as performance was unaffected by HDBR exposure and there was no HDBR/post-HDBR difference as measured by simple RT tasks (DeRoshia and Greenleaf, 1993; Lipnicki et al., 2009a), Choice/Complex RT task (Mahadevan et al., 2021), or the short version of the PVT (Basner et al., 2021), even though Lipnicki et al. (2009a) reported slower RTs along with decreased intra-individual variability, which they interpreted as improved executive functions. Similar to the space results, there was also no significant performance decrease during or after HDBR in any of the studies assessing DSST (DeRoshia and Greenleaf., 1993; Koppelmans et al., 2015; Lee et al., 2019; Basner et al., 2021; Pavy Le-Traon et al., 1994; Tays et al., 2022). Furthermore, even higher psychomotor speed was reported during HDBR (DeRoshia and Greenleaf, 1993), or a few days after HDBR with a greater increase in the non-exercise group than in the exercise groups (Koppelmans et al., 2015; Lee et al., 2019). In addition to the above described tasks administered in both space and HDBR studies, the following tasks were only assessed in HDBR: the Squares task (Pavy Le-Traon et al., 1994), the Counting oddball task (Mahadevan et al., 2021), the 3-stimulus oddball task (Brauns et al., 2021a), the Tapping task (DeRoshia and Greenleaf, 1993), and the Pattern comparison task (DeRoshia and Greenleaf, 1993; Pavy Le-Traon et al., 1994). In the Squares task, subjects must match the displayed squares to the predefined set of squares. In the Counting oddball task, subjects must count the rare occasions when a color changing stimulus box turns blue. The 3-stimulus oddball task includes three types of stimuli (frequent standard, infrequent irrelevant, and infrequent target) and should only press a button in case of infrequent target stimuli. In the Tapping task, subjects must tap as fast as possible with 1) two fingers, 2) using their preferred hand, or 3) using their non-preferred hand. In the Pattern comparison task, subjects are asked to decide whether two patterns are identical. While HDBR had no negative effect on performance in the Squares task, the Counting oddball task, the Tapping tasks, and the Pattern comparison tasks, Brauns et al. (2021a) reported impaired behavioral performance along with reduced amplitudes of two late Event Related Potential (ERP) components (P3a and P3b) during the last phase and after a 60-day HDBR exposure in the 3-stimulus oddball task.

Divided attention during spaceflight and HDBR was measured by Dual tasks where subjects had to perform 2 tasks simultaneously. The available studies show great variability regarding task choice. The majority of Dual task studies used a Tracking task, which was either combined with the Sternberg task (Manzey et al., 1995; Eddy et al., 1998; Manzey et al., 1998; Shehab et al., 1998), with RT tasks (Fowler et al., 2000; Bock et al., 2001; Bock et al., 2010), or with entering a 4-digit numerical code (Moore et al., 2019). The rest of the studies assessed a certain RT task with the Counting oddball task (Yuan et al., 2016; Lee et al., 2019; Mahadevan et al., 2021; Tays et al., 2021; Tays et al., 2022). As an index of Dual task performance, most studies measured Dual task cost or compared single task performance with Dual task performance (Manzey et al., 1995; Manzey et al., 1998; Fowler et al., 2000; Bock et al., 2001; Bock et al., 2010; Yuan et al., 2016; Lee et al., 2019; Mahadevan et al., 2021; Tays et al., 2021; Tays et al., 2022). Conversely, others only measured Dual task performance and did not make any comparisons with single task performance (Eddy et al., 1998; Shehab et al., 1998; Moore et al., 2019).

Dual task performance of astronauts was investigated in seven studies (Manzey et al. 1995; 1998; Eddy et al., 1998; Fowler et al., 2000; Bock et al., 2001; Bock et al. 2010; Tays et al., 2021; Moore et al., 2019). Regarding short-duration spaceflights, only the case study by Manzey et al. (1995) found impaired in-flight performance during the whole 8 days of the flight, while others did not report any performance deterioration (Eddy et al., 1998; Fowler et al., 2000; Bock et al., 2001). As for long-duration space flights, a case study by Manzey et al. (1998) found impaired Dual task performance to be limited to the first 2 weeks of in-flight while performance was intact during later in-flight sessions. In their half-year-long study with 3 astronauts, Bock et al. (2010) combined the Tracking task with four various RT tasks as dual tasks and found impairments to be limited to the Tracking task-rhythmic tapping RT task dual task combination which required complex motor programming (tasks are further described under Choice and Complex RT). Contrary, a study with 15 astronauts (Tays et al., 2021) found no space-related changes in Dual task performance. However, regarding the lack of clear performance deterioration, it is important to note that the first in-flight measurement point showed a high variance between subjects in Bock et al. (2010) (in-flight day 24 for one astronaut and in-flight day 106 for two astronauts), and the first in-flight measurement point was after 3 weeks in both studies (day 30 of in-flight for all subjects for Tays et al., 2021). Regarding readaptation, Moore et al. (2019) reported worse performance compared to pre-flight values only on the day of landing, while no further signs of post-flight performance decline were found in any of the studies (Manzey et al. (1995); Manzey et al. (1998); Moore et al., 2019; Tays et al., 2021; Manzey et al. (1995); Manzey et al. (1998) Fowler et al., 2000; Bock et al., 2001). Dual task performance under HDBR was measured by 1 short (16 days long) and 2 longer (60 and 70 days long) studies. One of the 2 long HDBR investigations measured both behavioral and fMRI parameters in 18 subjects and reported different aspects of their findings in 3 related articles (Yuan et al., 2016; Lee et al., 2019; Mahadevan et al., 2021). Regarding behavioral results, neither the short (Shehab et al., 1998), nor the long HDBR studies found impaired performance during or after HDBR exposure (Yuan et al., 2016; Lee et al., 2019; Mahadevan et al., 2021; Tays et al., 2022). Even though the behavioral data showed no impairments, fMRI results revealed increased HDBR-related brain activation during dual-tasking, which authors interpreted as an increased need for neurocognitive control during HDBR compared to pre- and post-HDBR (Yuan et al., 2016). It is important to note that studies showing impaired divided attention used a Tracking task as a dual task, while studies combining RT tasks with counting tasks did not find impaired performance. This may stem from the difference in task complexity and differences in cognitive load between the applied dual tasks.

Space-related changes in directed attention were only investigated in a 16 days long study (Eddy et al., 1998). They measured switching time between 2 tasks (a Mathematical task and a Manikin task). In-flight and post-flight switching time was worse than expected based on the learning curve in 2 of the 4 astronauts during the Mathematical task (when preceded by Manikin). Contrary, the only HDBR study using a similar paradigm found no alterations in directed attention during or after 16 days of HDBR (Shehab et al., 1998).

### Memory

The most frequently used paradigm to examine short-term and working memory in space and in HDBR was the Sternberg task (see Table 4). In this task, participants have to decide whether the displayed item on the screen is part of a memory set. Regarding space, the majority of the studies using this task were based on 8–16 days long short-durational space missions (Manzey et al., 1993; Manzey et al., 1995; Eddy et al., 1998; Newman and Lathan, 1999; Kelly et al., 2005), while only a case study was long-durational (Manzey et al., 1998). Clear in-flight performance decrement was only found by Kelly et al. (2005) with a modified Sternberg task, but contrary to the other available space studies, they analyzed Yes and No trials separately. They found that the RT differences between Yes and No trials were significantly increased as a function of the number of digits in the memory set during in-flight. Additionally, the long-durational case study by Manzey et al. (1998) also reported deteriorated performance in the smaller memory set version of the Sternberg task at some measurement points immediately before launch, and at the beginning of the in-flight period. However, performance started to improve immediately after launch and even went above the baseline level during later in-flight sessions. As for the pre-flight versus post-flight comparisons, this half-year-long case study was the only one finding worse performance a few days after return, which was still observed in the follow-up measurements (168 days after return).

In the 3 available 16–30 days long HDBR studies, subjects did not perform worse in the Sternberg task during or after HDBR exposure (DeRoshia and Greenleaf, 1993; Pavy Le-Traon et al., 1994; Shehab et al., 1998). DeRoshia and Greenleaf (1993) also investigated the effect of exercise in this task and reported significantly better performance in the exercise HDBR groups compared to the non-exercising HDBR group. Regarding pre- versus post-HDBR performance, once again no performance deterioration was reported in the above described 3 studies, although post-HDBR data were either not supported by statistical analysis or were not clearly reported.

Besides the Sternberg task, two additional tasks were administered to investigate space and HDBR related changes in short-term and working memory: the Continuous recognition memory task and the Running memory continuous performance task. In the former, stimuli are presented in a sequence (e.g., numbers: Eddy et al., 1998; Shehab et al., 1998; pictures: Friedl-Werner et al., 2020), and subjects have to compare the actual stimulus with the previous one. Eddy et al. (1998) reported no in-flight performance deterioration in the Continuous recognition memory task during a 16-days-long space mission. Similar to space results, behavioral performance showed no in-HDBR and post-HDBR deterioration in this task during a 16-days-long (Shehab et al., 1998) and a 60-days-long HDBR study (Friedl-Werner et al., 2020). Even though Friedl-Werner et al. (2020) found better performance on day 58 of the 60-day HDBR, they also reported increased BOLD signal in the hippocampal formation (left hippocampus and parahippocampal gyrus) in the non-exercising HDBR group compared to the exercising HDBR group, suggesting higher neuronal efficiency, thus a modulatory effect of training during HDBR. The Running memory continuous performance task was only assessed in one HDBR study (Seaton et al., 2009). In this task, subjects are instructed to decide whether a number displayed on the screen is the same as the immediately preceding one. Intact performance was reported during and after 60–90 days of HDBR.

Results of three additional memory tasks are presented in the following. The Probed recall memory, the Visual object learning task, and the Code memory delayed task are somewhat different from the previously presented short term memory tasks, as the recall time is significantly longer (or not detailed properly) in these tasks. The Probed recall memory was only administered in a short-durational space study (Dijk et al., 2001), while no HDBR studies are yet available. This task includes the presentation of six word pairs, one word pair in every 10 min, followed by a 1-min probed recall. Dijk et al. (2001) found declined in-flight performance in this task during 10–16 days long space missions of five astronauts. The Visual object learning task was used in a long-durational space study (Garrett-Bakelman et al., 2019) and in a HDBR study (Basner et al., 2021). In this task, subjects are instructed to memorize 10 sequentially presented 3D Euclidean shapes. Later they must decide whether objects sequentially presented on the screen are part of the memorized objects. While no in-flight deterioration was found in this task, Garrett-Bakelman et al. (2019) did report decreased post-flight performance after a one-year-long space mission. As for HDBR, Basner et al. (2021) found intact in-HDBR performance and no pre- to post-HDBR differences in a 60-days-long HDBR exposure. Similar to the results of Basner et al. (2021), no in-HDBR or post-HDBR behavioral performance decrement was detected in the Code memory delayed task (Seaton et al., 2009). In this task, subjects first have to memorize symbol-number pairs and then decide whether a symbol-number pair displayed on the screen was correct.

The effect of spaceflight and HDBR on spatial working memory was tested with the following tasks: the Line orientation memory task, the Array match to sample and Grid match to sample tasks, the Spatial location task. Regarding space, performance in the Line orientation memory task was investigated in a case study of a 6-day-long space mission (Benke et al., 1993a; Benke et al., 1993b) and in 2 half-year-long space missions (McIntyre et al., 2001; Takács et al., 2021). In this task, subjects are either instructed to compare or align the orientation of the actual line(s) with the earlier presented one. Takács et al. (2021) found significantly poorer performance in 5 astronauts during both the earlier (∼1.5 weeks) and later (∼1.5–2 months) stages of a half-year-long flight and even 2–8 days after return. ERP indicators showed changes similar to the behavioral data (reduced P3a and P3b amplitude), with the difference that while behavioral measures did return to pre-flight levels 2–3 weeks after landing, the ERP amplitudes did not. In contrast, no performance decrement was reported during or after the 6-days-long space mission for a single subject (Benke et al., 1993a; Benke et al., 1993b) or for 5 subjects participating in a half-year-long space mission (McIntyre et al., 2001). No HDBR study has yet assessed the Line orientation memory task. In the Array match to sample task, participants must decide which of the two 4 × 4 arrays of red and blue squares matches a previously displayed pattern. The Array match to sample task was only administered by the study of Moore et al. (2019), which found no pre- to post-flight performance difference immediately after a half-year-long mission. While this task has not been assessed under HDBR, a similar spatial working memory task, the Grid match to sample task was used by a HDBR study (Seaton et al., 2009), in which subjects have to decide which of the two grids were identical to the previous one. No HDBR related change in performance was detected in this task. Another spatial working memory task is the Spatial location task, where subjects must find the location of the letters on a grid presented subsequently based on a previously presented memory set. Performance in this task was only investigated during a 6-days-long space mission by a case study of Benke et al. (1993a, 1993b), reporting no change during and after spaceflight.

Spatial working memory involving mental rotation was explored by the following tasks during space missions and HDBR. In the Manikin task, participants are presented with stick figures with varying orientation. The figure is standing on a box containing either a rectangle or a circle symbol and holding various symbols in both of their hands. The task is to indicate which hand is holding the matching symbol. Performance in this task was measured in one 16-days-long space study (Eddy et al., 1998), and in 3 HDBR studies (DeRoshia and Greenleaf, 1993; Pavy Le-Traon et al., 1994; Shehab et al., 1998). While the 16-days-long space and HDBR studies found intact performance (Eddy et al., 1998; Shehab et al., 1998), the two 28–30 days long HDBR studies reported improved in-HDBR performance (DeRoshia and Greenleaf, 1993; Pavy Le-Traon et al., 1994). The only 2 studies analyzing pre- to post-HDBR differences found unchanged performance (DeRoshia and Greenleaf, 1993; Shehab et al., 1998). The Spatial matrix rotation task was used in a 16-days-long space study (Eddy et al., 1998), and in an equally long HDBR study (Shehab et al., 1998). The task was to decide whether each presented pattern (five illuminated cells in a 5 × 5 matrix) was either the same or completely different from as the previous one. Based on a learning curve, Eddy et al. (1998) reported worse performance than expected during and after the 16-day-long spaceflight in 1 out of 4 subjects. However, no description was provided regarding the post-flight data of the 3 other subjects. Shehab et al. (1998) obtained no significant changes during or after HDBR. The Mental rotation task was only investigated by two space studies (Matsakis et al., 1993; Leone et al., 1995). In this task, subjects must decide whether two 3D shapes displayed on the screen are rotated but identical, or mirror images. Both studies showed high individual variability regarding the duration of space missions (14–199 days for Leone et al., 1995; 1–6 months for Matsakis et al., 1993), and both found improved in-flight and post-flight performance. Unfortunately, related HDBR studies are not yet available.

Three additional tasks involving mental rotation were all administered in the same three studies: a half-year-long space study (Tays et al., 2021), a 60-days-long HDBR study (Tays et al., 2022), and a 70-days-long HDBR study, which was partially reported in three papers (Koppelmans et al., 2015; Cassady et al., 2016; Lee et al., 2019). In the Spatial working memory task, subjects are first instructed to form a triangle from three dots displayed on the screen (Probe stimulus), and then to decide whether three subsequently presented dots form a rotated version of the Probe triangle or not. Participants are also asked to perform a control task with three points appearing on the screen as a start, then they must decide whether a subsequently appearing dot is part of the three originally displayed ones. Tays et al. (2021) only provided pre- versus post-flight comparisons and reported no deterioration after a half-year-long mission in this task. As for the two available HDBR studies, no HDBR related performance change was obtained after 7 days (Lee et al., 2019), 30 days, or 60–70 days in HDBR (Lee et al., 2019; Tays et al., 2022), supporting spaceflight-related results of Tays et al. (2021). In Thurstone’s 2D card rotation task, subjects are first presented with a card with a 2D drawing of an abstract shape and then presented with new drawings. They must decide whether the new drawings are rotated or mirrored versions of the first drawing, or completely different. Like in the Spatial working memory task, unchanged performance was reported in this task after a half-year-long space mission (Tays et al., 2021) and during 60 days of HDBR exposure (Tays et al., 2022). The 70-days-long HDBR study even found improved performance during and after HDBR (Koppelmans et al., 2015; Cassady et al., 2016; Lee et al., 2019). Additionally, when comparing performance between the HDBR and the control group, Cassady et al. (2016) even found a greater in-HDBR improvement in the HDBR group. In the 3D cube rotation task, subjects were asked to decide which of the 2 cube assemblies on the screen was the rotated version of the cube assembly they had seen before. This was the only task where Tays et al. (2021) also included in-flight measurement points, but again, no changes were seen during or after a half-year-long mission (Tays et al., 2021) or during a 60-days-long HDBR exposure (Tays et al., 2022), while improved in-HDBR and post-HDBR performance was found in a 70-days-long HDBR study (Koppelmans et al., 2015; Cassady et al., 2016; Lee et al., 2019).

Long-term memory for face recognition was only investigated by de Schonen et al. (1998) in space. Their results showed that during (15 days to 6 months long) in-flight, astronauts’ performance for learning and recognition of faces learned-on-flight were significantly lower compared to learned-on-ground faces. Kelly et al. (2005) investigated learning new sequences during a 10-days-long space mission with the repeated acquisition of the Response sequences task, where astronauts had to learn a 10-response sequence and give an appropriate answer applying four response keys. They did not show any significant deterioration in learning new sequences, moreover they found a slight improvement in one of the performance indicators during in-flight and post-flight compared to pre-flight which could be attributed to learning effect. To our knowledge, no similar studies have been conducted under HDBR conditions.

### Executive functions

The effects of spaceflight and HDBR on executive functions were evaluated with the Stroop paradigm, the 2-back task, the Abstract matching task, the Balloon Analog Risk Task (BART), the Flanker task, and the Iowa Gambling task (see Table 5). Studies using the 1-back task are discussed under the Memory section. Versions of the Stroop paradigm were used by 2 short-duration space studies. In this task, subjects have to make a decision based on stimulus attributes which could be congruent or incongruent (the position and direction of the arrow in the study of Benke et al., 1993b; the printed color of words and the meaning of the word in the study of Pattyn et al., 2009) and then they have to press a corresponding key. Neither studies reported worse performance in-flight or after returning to Earth, and no HDBR studies using a Stroop paradigm are yet available.

The Abstract matching task, the 2-back task and the BART task were all administered in both space and HDBR. In the Abstract matching task, two pairs of objects are seen on the bottom left and bottom right corners of the screen varying on perceptual dimensions (e.g., color and shape). The target object is presented on the top center of the screen, and subjects must decide whether the target belongs more to the left or right bottom pairs, based on a set of implicit, abstract rules. In the 2-back task, participants are presented a sequence of stimuli one-by-one (e.g., fractals, numbers), and they must decide whether the current stimulus is the same as the one presented two trials earlier. As for the BART, subjects earn points with each button press that inflates a virtual balloon, however each pump action increases the risk of exploding the balloon and losing the points acquired in the trial. All three tasks were administered in a one-year-long space mission (Garrett-Bakelman et al., 2019) and a 60-days-long HDBR exposure (Basner et al., 2021). Both reported intact in-flight or in-HDBR performance, but Garrett-Bakelman et al. (2019) found decreased post-flight performance, while Basner et al. (2021) showed no post-HDBR deterioration. In addition to Basner et al. (2021), another 60-days-long HDBR study investigated executive functions with the 2-back task (Lipnicki et al., 2009a) and one 45-days-long HDBR study used the BART (Rao et al., 2014). Even though improved performance was found in the HDBR group during and after 60 days HDBR exposure in the 2-back in the study of Lipnicki et al. (2009a), authors also reported diminished practice effect compared to the control group. However, the 2 groups were very different in sample size, age, and gender distribution. Rao et al. (2014) found unchanged risk-taking behavior during and after 45 days of HDBR compared to baseline in the BART, while pre- to post-HDBR comparisons for fMRI measurements revealed diminished differentiation of winning and losing in the prefrontal cortex. It should be noted however, that diminished sensitivity might be a result of habituation after performing the same task for 8 sessions.

The Flanker task and the Iowa Gambling task were only administered in a 60-days-long HDBR study of Lipnicki et al. (2009a; 2009b). In the Flanker task, 5 arrows appear on the screen. The middle one is pointing to either the same or the opposite direction as the others. Subjects must press a button according to the direction of the middle arrow. In the Iowa Gambling task, participants must select one card out of four virtual decks of cards. They either win or lose game money with each card selection, however, the reward versus penalty cards balance is different for each deck of cards. Participants are informed that there are worse decks which should be avoided to win as much money as possible. In the Flanker task, Lipnicki et al. (2009a) found similar results to the 2-back task, as while improved performance was found in the HDBR group during and after 60 days of HDBR exposure, diminished practice effect was also present compared to the control group. As for the Iowa Gambling task, the authors used another approach: half of the subjects completed the task during a pre-HDRB and an in-HDBR session, while the other half completed an in-HDBR and a post-HDBR session. Results show no changes in performance between pre- and in-HDBR sessions, but improved performance between in- and post HDBR sessions. The difference between the 2 groups was further analyzed by Lipnicki et al. (2009b), where they compared the first measurement points of the same 2 groups. The authors reported equal performance in the 2 groups, but a failure to develop an adaptive strategy as the task progressed in the in-HDBR group.

### Reasoning and mathematical processing

Relatively few studies have investigated reasoning and mathematical processing in space and HDBR (see Table 6). Two reasoning tasks have been administered in this field. In the Grammatical reasoning task, sentences describing the order of symbols/letters are presented together with a certain set of symbols/letters. Subjects must evaluate whether the sentences are true for the presented symbols/letters. Grammatical reasoning task performance was investigated during short (Manzey et al., 1993) and long-duration space flights (Manzey et al., 1998). Neither of them showed in-flight deterioration (Manzey et al., 1993; Manzey et al., 1998). Moreover, Manzey et al. (1993) observed improved performance after a 8-days-long space flight. While Manzey et al. (1998) found no changes comparing pre-flight and post-flight performance, they did report performance improvement in a later follow-up assessment (day 168 of post-flight) of a 438-days-long flight. In the Matrix reasoning task, subjects are shown a series of patterns displayed on a grid. One element of the grid is missing, and subjects must decide which of the presented patterns fits the missing part of the grid. Similar to space results for the Grammatical reasoning task, Garrett-Bakelman et al. (2019) reported intact in-flight Matrix reasoning performance during the one-year-long mission, however declined performance was described after flight. Both reasoning tasks have also been assessed under HDBR (DeRoshia and Greenleaf, 1993; Pavy Le-Traon et al., 1994; Basner et al., 2021). No clear deterioration was detected in any of the HDBR studies. Regarding Grammatical reasoning, subjects showed improved performance during HDBR (DeRoshia and Greenleaf, 1993; Pavy Le-Traon et al., 1994), but no pre-HDBR post-HDBR differences. While DeRoshia and Greenleaf (1993) also reported significantly greater improvement in case of the non-exercising HDBR group compared to HDBR groups which exercised regularly as a countermeasure. Additionally, Basner et al. (2021) found intact performance during and after HDBR in the Matrix reasoning task.

Mathematical processing was investigated by one 16-days-long space study (Eddy et al., 1998), and 2 HDBR studies (Shehab et al., 1998; Seaton et al., 2009). In the Mathematical processing task, subjects have to add or subtract 3 single-digit numbers and indicate whether the result is greater or less than five. Eddy et al. (1998) found in-flight and post-flight performance degradation in 2 out of 4 astronauts in the Mathematical processing task, indicating worse than expected performance based on a learning curve. However, no description was provided regarding post-flight data for the 2 subjects showing no changes. Both HDBR studies reported performance to be unchanged both during and after the 16-days-long (Shehab et al., 1998) or 60–90 days long HDBR exposures (Seaton et al., 2009).

### Cognitive processing of emotional stimuli

In this section, we only discuss results related to cognitive processing of emotional stimuli (see Table 7), while self-report questionnaires reflecting emotional states are outside the scope of this review. Only two studies targeted emotional processing during spaceflight: one assessed the Emotional Stroop task (Pattyn et al., 2005; Pattyn et al., 2009), the other used the Facial emotion recognition task (Garrett-Bakelman et al., 2019). The Emotional Stroop task was measured before, during, and after a 11-days-long short-durational space mission. This task is a version of the classic color-word Stroop which includes general and mission specific words. Results showed higher error rates during in-flight, but not after returning to Earth (Pattyn et al., 2005; Pattyn et al., 2009). Facial emotion recognition was evaluated by the Emotion Recognition task of the Cognitive test battery (Basner et al., 2015) in a twin study of a one-year-long space mission (Garrett-Bakelman et al., 2019). In this task, subjects are presented with pictures of emotional facial expressions of varying types and intensities, and they must select an emotion label correctly describing the expressed emotion (happy, sad, angry, fearful, or no emotion). While results showed intact in-flight emotion recognition, they reported decreased post-flight performance.

As for HDBR, four studies are available in this subject, and only Basner et al. (2021) used an emotional processing task which was also administered in space. This 60-days-long HDBR study also used the Emotion Recognition task and reported decreased performance with increasing time spent in HDBR, and worse post-HDBR performance compared to baseline (Basner et al., 2021). The three other HDBR studies investigated cognitive processing of emotional stimuli with tasks that have not been used in space studies. Brauns et al. (2019) assessed the Affective picture evaluation task along with concomitant EEG recordings after a 30-days-long HDBR exposure and compared data with control subjects. In this task, unpleasant, pleasant, and neutral pictures are presented in a random order, and subjects are asked to rate the arousal and the valence of their emotional perception in each trial. There were no differences between the two groups in behavioral results. Concerning ERP, while neutral pictures elicited similar ERP responses (P300 and LPP) in both groups, emotional stimuli evoked smaller ERP components in the HDBR group compared to the control group.

Attention to emotional faces was investigated by two HDBR studies so far. One used the Emotional Flanker task (Liu et al., 2012) during a 45-days-long HDBR exposure, while the other used the Dot-probe task during a 15-days-long HDBR exposure (Jiang et al., 2022). In the Emotional Flanker task, subjects are presented with three pictures including happy, neutral, and fearful faces in each trial and instructed to pay attention to the center target picture regardless of the other two pictures and to press a given button according to the emotional valence (happy, neutral, or negative) of the target picture as quickly as possible. Liu et al. (2012) obtained poorer performance in this task during 45 days of HDBR but found no significant pre- to post-HDBR differences. Jiang et al. (2022) investigated the effects of 15 days of HDBR on attention bias to threatening stimuli with the Dot-probe task. To test attention allocation to emotional stimuli with this task, subjects are presented with a pair of pictures showing angry and neutral faces appearing either the left or the right side of the screen followed by a triangular shape appearing either side of the screen. Subjects are instructed to press a button only if the triangular shape is facing upwards (Go stimulus) and press no buttons when it is facing downwards (NoGo stimulus). Results show that compared to pre-HDBR, response speed was significantly slower to threat stimuli than to neutral stimuli in the middle of HDBR (on day 8 of HDBR), than that of neutral stimuli in the middle of bedrest (on day 8 of HDBR), which is suggested to reflect attentional avoidance of threatening stimuli. However, there was no difference in reaction time between pre-HDBR and any other measurement points during or after HDBR.

## Discussion

Taken together, results of space and HDBR studies are far from being conclusive in any of the revised seven cognitive categories. No clear performance impairment was reported during space missions and HDBR exposure by the majority of the studies. Results regarding sensorimotor skills are conflicting. Decreased performance was observed in tasks requiring sensorimotor skills (tracking tasks, and Pointing arm movement task) in space. Decreased sensorimotor speed in the Motor praxis task was only found in HDBR, while the only space study showed intact performance. Even though pre-flight and in-flight comparisons have not yet been investigated for manual dexterity and fine motor control, the available studies consistently reported decreased post-flight performance during the early post-flight measurements. More investigations are needed to find out whether bimanual coordination is challenged by space missions, even though HDBR studies found no performance decrement. Regarding time estimation, only one of the three space studies found significant change, which was in parallel with the results of the only available HDBR investigation. Most behavioral results showed intact performance during simple tasks targeting psychomotor speed and attention during both short- and long-duration space flights. Space-related performance deterioration was revealed in the Random switching task, some cases with the Dual-task, and in one case with the Simple RT task. Astronauts’ performance showed no pre- to post-flight changes, except for a case study, which found post-flight impairment in the PVT and in the DSST. As for HDBR studies, in-HDBR and post-HDBR performance decrement was observed in the 3-stimulus oddball task, and post-HDBR decrement was found in Simple RT task by one study. Concerning memory, the majority of studies show no impairments, except for few tasks in-flight (Spatial matrix rotation, Probed recall memory, the Sternberg in one study, the Recognition of new faces, and the Line orientation memory) and post-flight (the Visual object learning, the Spatial matrix rotation, the Sternberg in one study, and the Line orientation memory). In addition, no memory task showed performance deterioration during or after HDBR. Regarding executive functions, studies found no performance change in space and only one case study found post-flight impairment in three memory tasks. Similarly, most research did not report worse executive performance during or after HDBR, but diminished practice effect was detected in some executive function tasks and altered decision making was found in the Iowa Gambling task, and in the BART. Likewise, no clear space- or HDBR-related performance decrement was found regarding reasoning and mathematical processing. As for cognitive processing of emotional stimuli, although both space and HDBR results show worse performance in this category, space research is too scarce to draw a clear conclusion.

As this review is aiming at addressing the question whether HDBR studies are adequate analogs for spaceflight induced cognitive changes, in the following, we discuss factors contributing to diversity of the results and challenging the comparability between studies.

### Duration of space and head-down tilt bedrest studies

Cognitive performance during space missions has been investigated for decades. The oldest studies in this review are almost 30 years old. While the majority of older studies are based on short duration missions, more and more long-term space studies have been published in recent years. The shortest spaceflight included in this review was only 6 days long (Benke et al., 1993a; 1993b) and the longest was 438 days long (Manzey et al., 1998). The typical length of flights was between 8–16 days for short duration studies (e.g., Eddy et al., 1998) and half year for long duration missions (e.g., Tays et al., 2021). Concerning HDBR, the shortest HDBR exposure included in this review was 15 days long (e.g., Jiang et al., 2022), while the longest HDBR exposure was 90 days (Seaton et al., 2009). The reviewed HDBR studies were typically 28–70 days long (e.g., Tays et al., 2022). The significant difference in time frame between space and HDBR studies limits the compatibility between these conditions. It is also important to mention that the exact duration of the space mission and the timing of data acquisition for each astronaut can show a great variability in some of the space studies (e.g., Bock et al., 2010), while the schedule for HDBR studies are fixed across subjects for each study.

### Baseline measurements

The studies described in this review used diverse baseline measurement points including a high variability regarding the timeframe before launch. Pre-flight measurements were scheduled at least 1.5–2 months before launch in some studies (e.g., Moore et al., 2019), others added a baseline measurement closer to the flight, even 1–2 weeks before the flight (e.g., Benke et al., 1993a), while, very rarely, there are studies with baseline measurements only within 1 week before the flight (e.g., Manzey et al., 1993). Timing of the pre-flight measurement point(s) seems crucial as some of the studies found differences in performance between earlier and later pre-flight measurements (Manzey et al., 1998; Pattyn et al., 2009). In contrast, a case study of Manzey et al. (2000) found no differences in a Tracking task between 2 months before launch with 1–3 weeks before launch. These results highlight the importance of the timing and frequency of pre-flight data acquisition in interpreting performance in space.

In HDBR studies, the timing of the baseline measurements show less variability compared to space studies, as baseline measurements are generally scheduled within 1–2 weeks before HDBR despite using single (e.g., Yuan et al., 2016) or multiple measurement points (e.g., Basner et al., 2021). It is also worth noting that several studies included pre-flight practice session(s) (e.g., Tays et al., 2021), and pre-HDBR practice session(s) (e.g., Yuan et al., 2016). The inclusion of practice sessions along with the timing and frequency of pre-flight and pre-HDBR measurement(s) may influence the learning effect for the given tasks which may also impact the results.

### In-flight and in-HDBR measurements

There is also a wide variation in both in-flight and in-HDBR measurements, which may also challenge the interpretation of the results. For 1 or 2-week long shuttle flights, the first in-flight measurements were often performed only a few hours after take-off (e.g., Bock et al., 2001). In long-duration space missions, the timeframe for the earliest in-flight measurements points varied between 4–12 days (e.g., Takács et al., 2021) to even 1 month or beyond after take-off (e.g., Tays et al., 2021), while some studies had no in-flight measurements at all (e.g., Moore et al., 2019). Regarding HDBR, the first in-HDBR measurements were either scheduled at the first 1–2 days (e.g., Basner et al., 2021), after the first week (e.g., Yuan et al., 2016), or rarely even after a month in-HDBR (Tays et al., 2022).

### Post-flight and post-HDBR measurements

Post-flight and post-HDBR measurements also show high variability including timing and frequency. In most space studies, all post-flight measurements were within 1–3 weeks after return to Earth (e.g. Moore et al., 2019); while some studies included post-flight measurements even after 2 months (e.g., Tays et al., 2021). Regarding the timing of the first post-flight measurements, it was either scheduled within a few hours (e.g., Moore et al., 2019) or a few days after return (e.g., Takács et al., 2021). In the vast majority of HDBR studies, all post-HDBR measurements took place within 2 weeks, except for the study of Lipnicki et al. (2009a). Concerning HDBR, some studies scheduled their earliest post measurement points as early as day 1 (e.g., DeRoshia and Greenleaf, 1993), while other studies had the earliest measurements as late as week 1 post-HDBR (e.g., Yuan et al., 2016) or even a month post-HDBR (Lipnicki et al., 2009a).

Regarding the choice and interpretation of the first in-flight and post-flight measurements, it is worth noting that many astronauts - especially novices - suffer from SMS up to 4 days in mission and to a lesser extent after return (Kanas & Manzey, 2008). SMS is associated with serious malaise, which can adversely affect the outcome of cognitive experiments. Therefore, merging such early measurements with later data points may lead to the false impression of a generalized deficit.

### Methodological differences

In addition to the above described variability regarding timing and frequency of the data acquisition, other methodological variations among studies may further encumber solid interpretation of the results. Such differences between studies may arise from the following factors: 1) task-based differences, 2) differences between calculated performance indicators, and 3) differences in the applied statistical methods.

1. Studies also show a great variability in task choice, which makes results less comparable. Even in the rare cases where the same task is used to measure a particular function, there are remarkable discrepancies. A great example for this issue is the numerous versions of the set of studies using the Sternberg task assessed by the available space and HDBR studies. While some of these studies only administered a larger (4-letter long) memory set (Eddy et al., 1998; Shehab et al., 1998), other studies used both smaller (2-letter long) and larger (4-letter long) memory sets as a separate task (Manzey et al., 1993; 1995; 1998), in some of the studies, the length of the memory set varied between 1 and 6 (Kelly et al., 2005), or 1 to 7 (Newman and Lathan, 1999). In Pavy Le-Traon et al. (1994), the size of the memory set is not even clear.

2. On top of huge variability in task choice, there are various performance indicators used across studies, even when using the same or similar tasks. For example, even though a relatively large set of studies administered tracking tasks, they also show high variability regarding the chosen performance indicators (e.g., Eddy et al., 1998; Manzey et al., 1998; Fowler et al., 2000). This is also true for Dual task studies, which may greatly complicate the interpretation of the results. Some studies calculated a specific value for the difference between dual and single tasks (e.g., Tays et al., 2021; 2022), some others looked at the difference between the two tasks (e.g., Manzey et al., 1995; 1998) in one analysis, while some others only analyzed dual task performance (e.g., Moore et al., 2019).

3. The statistical methods of the reviewed studies are also diverse. Some of the investigations - mainly short-term space flight case studies - included numerous measurement points where some of the successive data points showed different performance outcomes, which makes interpretation difficult. For example, even though Manzey et al. (2000) reported more tracking errors during a 3-weeks-long flight, only one half of their measurements differed significantly from the baseline. Certain papers report performance changes without specifying which measurement points were different (e.g., Dijk et al., 2001), while the twin study of Garrett-Bakelman et al. (2019) does not report differences between pre- and in-flight per task and compares a single astronaut’s performance to his twin brother, which makes results less comparable with other long-durational space studies. In addition to the fact that statistical analyses may vary from paper to paper, the presence or lack of alfa corrections for multiple comparisons also affects the interpretation of the results. For example, even though Koppelmans et al. (2015), Cassady et al. (2016), Lee et al. (2019), Mahadevan et al. (2021), and Yuan et al. (2016) describe different aspects of the same research, behavioral data were only corrected for multiple comparisons in some of these articles.

A recent report of a NASA-ESA expert group (Seidler et al., 2022) suggested the administration of standardized core measurements in both space and space analog studies to gain more insight into the effect of spaceflight on human brain, eye, and behavior. Therefore, they suggest including the same set of ocular measurements, up-to-date MRI protocols, cognitive and operational performance measurements, along with sensorimotor measures in future studies. For standardized cognitive measurements, Seidler et al. (2022) recommend NASA Cognition test battery (Basner et al., 2015; Moore et al., 2017), the ROBoT-r tasks (Ivkovic et al., 2019), along with the flight-certified Spatial Cognition test battery to be assessed “at least twice pre-flight, once early in-flight, once mid in-flight, once late in-flight, and twice post-flight. The pre- and postflight performance assays should be performed on the same day, or as near to the time of the MRI scan as possible, to support the interpretation of the functional and structural imaging data.”

### Control variables

Performance in cognitive tests is highly dependent on one’s actual physiological and psychological state, such as stress, mental fatigue, and sleepiness. Therefore, the influence of these factors should be considered as control variables when assessing cognitive performance. This is particularly important in spaceflight conditions when mission related requirements like time shifts, arrival of cargo, and extravehicular activity could grossly interfere with ongoing life science experiments. These influences could be statistically controlled for or at least discussed, provided that control variables reflecting these conditions are measured and made available to researchers. Jones et al. (2022) used visual analog scale ratings to assess sleep quality, workload, stress, mental fatigue, physical exhaustion, tiredness, and sleepiness and publicly available mission timelines to assess sleep shifts. They found a statistically significant relationship between sleep time and psychomotor speed. The effects of workload and fatigue on performance were investigated in other studies described in this review. Results of Manzey et al. (1998); Manzey et al. (2000), and Eddy et al. (1998) all suggest higher subjective workload and/or fatigue to be associated with performance decrement.

Individual differences should also be accounted for, particularly in space studies with a small number of subjects. Most importantly, considerable individual differences in sensitivity to headward fluid shift seem to exist. In a HDBR study, Lee et al. (2019) analyzed separately the 5 subjects who developed SANS (based on the extent of optic disc edema) and the six subjects without SANS and found increased dependency on visual cues in SANS subjects. The authors cite their own earlier data showing that only about a 1/3 of astronauts manifested SANS (Lee et al., 2016). These observations may explain the null results in some HDBR and space experiments.

EEG data can help to objectively assess sleepiness, which may be an important explanatory factor. Unfortunately, existing data do not provide a consistent picture. Brauns et al. (2021b) found a decrease in HDBR in all frequency bands, which is somewhat consistent with Cebolla et al. (2016) who obtained a decrease in the alpha band in a space experiment. This is in contrast with the results of Chéron et al. (2006), where they report increased alpha in space. Petit et al. (2019) also found an increase in space, but they analyzed the theta band in particular, which may be an indicator of increased sleepiness.

### Physiological, psychological, and situational differences between spaceflight and head-down tilt bedrest

HDBR is an adequate ground-based model for simulating many of the physiological symptoms of weightlessness, including cardiovascular changes (Pavy Le-Traon et al., 2007; Barbic et al., 2019), and cephalic fluid shift (Hargens and Vico, 2016; Koppelmans et al., 2017), and volumetric gray matter changes (Koppelmans et al., 2017). Beyond the gross similarities, such as restricted mobility and privacy, monotonicity, total control of nearly all vital functions by the organization as a total institution, etc., there are some physiological, psychological, and situational differences between spaceflight and HDBR.

Regarding physiological discrepancies, altered gravitational conditions result in different somatosensory inputs, which is an important difference between actual and simulated space missions primarily in terms of spatial orientation. On the other hand, it also changes the geometry of the brain and CSF relocation, which can influence the results of EEG studies (e.g., Brauns et al., 2021a). Additionally, space missions and HDBR may impact the vestibular system differently. While microgravity does induce structural and functional changes at multiple stages of vestibular processing (Carriot et al., 2021), little is known about how vestibular processes are affected by HDBR under normal gravity conditions. Nevertheless, during HDBR, the altered body position may indirectly affect vestibular processing due to the sensory change resulting from axial body unloading (Yuan et al., 2018). It is also worth noting that some physiological stressors of the space environment are not present in HDBR. High level of ambient noise, for example, seems to be an unavoidable stressor in space, owing to the need for increased demand for cooling electrical components. Elevated noise levels can lead to elevated arousal, fatigue, and diminished attention. Altered space-related circadian rhythm and consequent sleep disturbance may also be present in HDBR studies with variable extent, therefore sleep monitoring as a standard control measurement in both space and simulation studies would help to compare and interpret results.

Concerning the psychological analogy between HDBR and spaceflight, the first remark is that both involve the whole person, not just their cognitive or other functions. HDBR is one of the many forms of terrestrial space analog environments and situations: it is a man-made Extreme and Unusual Environment (Suedfeld, 2012, Suedfeld, 2018). The psychology of HDBR is related to sensory deprivation, isolation, and confinement, which is also true for spaceflight (e.g., Suedfeld et al., 1964; Suedfeld, 1975). However, there is significant divergence between subject selection for space missions and for HDBR experiments, which can also lead to differences in motivational background of the selected subjects. Astronauts are highly trained subjects undergoing extremely competitive selection phases before getting the opportunity to join the space crew of an actual space mission. While HDBR subjects are usually also selected based on detailed medical and psychological screening before being exposed to weeks of HDBR for research purposes, their motivational background might be different from space crew members. While it is suggested that highly trained and motivated astronauts may be able to compensate for spaceflight-induced deficits in cognitive performance (Basner et al., 2015), no study is yet available to investigate the role of motivational differences between these conditions on cognitive performance.

Situational differences between the two conditions should also be considered. Unlike HDBR personnel, the weeks and months before a mission can be extremely busy for astronauts, who are scheduled every minute to travel from one space center to another, often battling jet lag, while also taking baseline measurements for experiments. This is demonstrated by Jones et al. (2022) reporting suboptimal sleep, high perceived workload, and stress levels during pre-flight, which might be a reason for space studies not finding deteriorations in-flight compared to pre-flight. Compared to HDBR, a higher level of workload and physical activation is also present during space missions. While astronauts lead active lives with mandatory daily exercise, HDBR volunteers are confined to bed with minimal movement unless required by countermeasures. Regarding cognitive performance presented in this review, the majority of the HDBR studies comparing cognitive performance in an exercise countermeasure HDBR group with a no-exercise HDBR group reported no group differences (Koppelmans et al., 2015; Mulavara et al., 2018). Nevertheless, differences in workload, activity, sleep, and stress level between actual spaceflight and HDBR conditions should be considered when comparing results between these situations.

## Conclusion

The remarkable variations in sample size, methodology, and statistical analyses limits the interpretation and comparability of the reviewed space and HDBR studies. Regarding the seven cognitive categories reviewed in this study, most studies found no clear performance impairment during and after space missions or HDBR exposure, and results of the space and HDBR studies are not even showing parallel results in some cases. The predominance of results that show no deterioration or even improvement may be explained by the fact that the astronauts’ workload, stress levels and sleep time in the pre-flight reference period may be no better than that during the spaceflight (Jones et al., 2022). Practice effects can also mask performance degradation relative to baseline and might even manifest as improvement in the recovery period. In addition, medication may also impact cognitive performance. While astronauts are allowed to take medications during their missions, individual medical information is often not available for research purposes due to ethical reasons and therefore, cannot be controlled for.

As for the question, whether HDBR studies capture the true nature of spaceflight induced cognitive changes, the above detailed methodological issues still need to be addressed. Standardized study protocols and standardized core measurements suitable for both real and simulated space flight conditions still need to be developed and standardized statistical methods should be used to allow comparisons between studies. Until then, this question remains open.

Possible future direction to increase compatibility between space and HDBR studies are to include backup astronauts or astronaut candidates as a HDBR control group and to mirror the timing of data acquisition of the astronauts in the space group as closely as possible. Finally, it is also advisable to include an exercise regime comparable to that of astronauts for all HDBR studies focusing on countermeasures other than exercise itself.
